# Breast Cancer Risk Modification in Women with Pathogenic Variants in *BRCA1*, *BRCA2*, *ATM*, *CHEK2*, and *PALB2*

**DOI:** 10.1158/2767-9764.CRC-24-0592

**Published:** 2025-05-12

**Authors:** Allison W. Kurian, Elisha Hughes, Ryan Bernhisel, Eudora Hu, Eric C. Polley, Siddhartha Yadav, Chunling Hu, Jennifer L. Caswell-Jin, Esther M. John, Aladdin H. Shadyab, Rowan Chlebowski, Rami Nassir, Peter Kraft, Marcia L. Stefanick, Fergus J. Couch

**Affiliations:** 1 Department of Medicine, Stanford University School of Medicine, Stanford, California.; 2 Department of Epidemiology and Population Health, Stanford University School of Medicine, Stanford, California.; 3 Myriad Genetics, Salt Lake City, Utah.; 4 Department of Public Health Services, University of Chicago, Chicago, Illinois.; 5 Mayo Clinic, Rochester, Minnesota.; 6 Division of Geriatrics, Gerontology, and Palliative Care, Department of Medicine, Herbert Wertheim School of Public Health and Human Longevity Science, University of California San Diego, La Jolla, California.; 7 The Lundquist Institute, Torrance, California.; 8 Department of Pathology, School of Medicine, Umm Al-Qura University, Makkah, Saudi Arabia.; 9 National Cancer Institute, NIH, Rockville, Maryland.

## Abstract

There are limited prospective data on whether established risk factors modify breast cancer risk in women with pathogenic variants (PV) in *BRCA1/2* and virtually no risk modification data for *ATM*, *CHEK2*, or *PALB2*. We conducted a nested case–control study in the Women’s Health Initiative (WHI), randomly selecting women with and without breast cancer for DNA sequencing. We evaluated breast cancer odds associated with obesity, family cancer history, smoking, alcohol, parity, breastfeeding, oophorectomy, tubal ligation, neighborhood socioeconomic status, and menopausal hormone therapy (MHT) with estrogen and progestin or estrogen only in PV carriers and noncarriers. In exploratory analyses, we grouped genes by established predisposition for estrogen receptor (ER)-positive (*ATM* and *CHEK2*) or ER-negative (*BRCA1 *and* PALB2*) disease. Multivariable models with interaction terms were used to assess differential risk modification by PVs. Among 12,957 WHI participants, 287 carried PVs. Breastfeeding was modestly associated with reduced risk for *PALB2* [OR = 0.08; 95% confidence interval (CI), 0.00–0.92; *P* value = 0.042]. With one-sided 95% CI, power was sufficient to exclude OR ≥2.0 with obesity for *ATM* and *BRCA2*; smoking and alcohol for *CHEK2*; no breastfeeding for *ATM*; no oophorectomy for *BRCA2* and *CHEK2*; no tubal ligation for *CHEK2*; and neighborhood socioeconomic status for all genes. Estrogen + progestin MHT was modestly associated with increased risk for ER-positive PVs (OR = 7.31; 95% CI, 1.14–64.20; *P* = 0.036). PVs did not modify risk (interaction *P* ≥ 0.05). *BRCA1/2*, *ATM*, *CHEK2*, and *PALB2* PV carriers do not have breast cancer OR ≥2.0 with many established risk factors. However, MHT warrants additional study in PV carriers.

**Significance:**

There is limited information on whether established risk factors increase breast cancer risk from PVs. In the WHI, PV carriers had no substantial (≥2-fold) increase with most risk factors, except potentially MHT in *ATM* or *CHEK2* carriers. The results may inform counseling and research on MHT.

## Introduction

Genetic testing for inherited cancer risk is indicated for most patients with breast cancer (1). Genetic testing results affect cancer treatment, secondary cancer risk reduction, and care of family members. Pathogenic variants (PV) are commonly found in *BRCA1*, *BRCA2* (*BRCA1/2*), *ATM*, *CHEK2*, and *PALB2* (2, 3). We and others have reported on the prevalence and penetrance (associated cancer risk) of PVs in these genes among women who were not selected based on strong family cancer history or early diagnosis age (4, 5).

For individuals who inherit PVs in breast cancer–associated genes, it is important to know whether potentially modifiable exposures, such as menopausal hormone therapy (MHT) or alcohol use, substantially increase breast cancer risk. Prior studies investigated risk factors, including smoking, alcohol consumption, parity, breastfeeding, physical activity, body mass index (BMI), diagnostic and therapeutic radiation exposure, oral contraceptives, and MHT, among carriers of *BRCA1/2* PVs (6–21), but many were retrospective and few included *ATM*, *CHEK2*, or *PALB2*. We leveraged the Women’s Health Initiative (WHI), a prospective observational study and randomized clinical trials that enrolled postmenopausal women. We report on penetrance and breast cancer risk–modifying factors for PVs in *BRCA1/2*, *ATM*, *CHEK2*, and *PALB2* among WHI participants.

## Materials and Methods

### Study aims

The primary objective was to evaluate the association of established risk factors with breast cancer risk among carriers of inherited PVs. As a secondary objective, we tested whether established factors modify risk differently for PV carriers versus noncarriers.

### Study sample

The WHI (RRID: SCR_011789) is a prospective study of morbidity and mortality among postmenopausal women, including clinical trials and an observational cohort. Details of the WHI study design, conduct, and follow-up were published previously (22–24). WHI participants were of ages 50 to 79 years, postmenopausal, had life expectancy of at least 3 years, and were enrolled throughout the United States from September 1993 to December 1998.

This study combined two WHI ancillary studies (AS), AS508 and AS551 (AS551 is a component of the CARRIERS consortium). Details of AS508 and of the CARRIERS study were published previously (4, 5). Both ASs included women without history of breast cancer at WHI enrollment, who either were diagnosed with invasive breast cancer or were comparison women who remained cancer-free as of September 20, 2017. For both AS508 and AS551, women with incident breast cancer and cancer-free comparison women were randomly selected from all WHI participants with a banked DNA sample.

All incident breast cancers were centrally adjudicated, and participant demographic, clinical, and family history data were collected as previously described (22–24). WHI variables used in this study were age at enrollment, race, and ethnicity [according to previously published WHI categories (25)], BMI (underweight, <18.5 kg/m^2^; normal, 18.5–24.9 kg/m^2^; overweight, 25–29.9 kg/m^2^; and obese, ≥30 kg/m^2^), breast cancer diagnosis (yes/no), family history of breast cancer in first-degree relatives (yes/no), cigarette smoking (ever use: yes/no), alcohol consumption (ever use: yes/no), parity status (parous/nulliparous), breastfeeding history (ever/never), oophorectomy (yes/no), tubal ligation (yes/no), neighborhood socioeconomic status [NSES; a numeric variable used in prior WHI research (26)] and MHT use [ever use: yes/no, for estrogen and progestin (E + P) and estrogen only (E-only)]. For MHT use, we distinguished between participants in the WHI clinical trials versus observational study. If a participant was randomized to the placebo arm of either the E + P MHT clinical trial or the E-only MHT clinical trial, we used that participant’s self-reported data to categorize her MHT use for analyses.

### Genetic sequencing

Sequencing for AS508 was performed by Myriad Genetics and for AS551 by the Couch Laboratory at Mayo Clinic, as previously reported (4, 5). For AS508, next-generation sequencing (NGS) was performed with sequence and large rearrangement (LR) analysis for all genes. LR testing for deletions and duplications was successfully performed by NGS dosage analysis on most samples. Portions of noncoding intronic regions surrounding the exons were analyzed by NGS. Long-range PCR was used for initial amplification of *CHEK2* gene regions to avoid well-characterized pseudogenes. Samples were pooled and loaded onto massively parallel NextGen sequencers for 2 × 150 base paired-end reads. The minimum coverage depth used for sequence determination by NGS was 50× per base. AS551 sequencing was performed using a custom amplicon-based QIAseq panel. Target regions were exons <150 bp with 10 bp of flanking intronic sequence. Samples were pooled in batches of 768 and loaded onto massively parallel NextGen sequencers for 2 × 150 base paired-end reads. The minimum coverage depth used for sequence determination was 20× per base with a minimum alternative allele frequency of 0.20. LRs were identified by PatternCNV (27).

### Variant classification

Variant classification followed criteria of the American College of Medical Genetics and Genomics and the Association for Molecular Pathology (28). Variants were classified as PV or likely PV if (i) they conferred a truncation, loss of select initiation codon, or splice donor/acceptor effect; (ii) functional data demonstrated an effect on protein function relevant to disease phenotype; or (iii) pathogenicity was otherwise demonstrated in the literature. If no significant functional data were available, then missense, silent, and intronic variants were classified as variants of uncertain significance (VUS), likely benign or benign based on modified American College of Medical Genetics and Genomics guidelines and according to standard laboratory procedures. The *CHEK2* I157T variant was not classified as pathogenic, consistent with practice guidelines.

### Statistical analysis

All analyses were conducted using R version 4.1.3 and independently reproduced using R version 4.3.2. We assessed the penetrance of PVs (defined as the magnitude of breast cancer risk associated with PV carriage) in each gene and breast cancer risks associated with clinical factors in terms of ORs, two-sided 95% confidence intervals (CI), and two-sided *P* values from multivariable logistic regression models with the Firth penalized likelihood. Additional details regarding the coding of variables are provided in Supplementary Methods S1.

To evaluate gene penetrance, we constructed a separate logistic regression model for each gene. Breast cancer status was the dependent variable. Independent variables included PV status, age at WHI enrollment, and AS (AS508 or AS551). Women with PVs in breast cancer–associated genes other than the gene of interest were excluded, as were women with VUS in the gene of interest, unless the VUS co-occurred with a PV in the same gene.

For the primary analysis, we evaluated breast cancer risks associated with established risk factors in terms of odds ratios (ORs) and two-sided 95% CIs from logistic regression models constructed separately in subcohorts of women who tested negative for PVs (noncarriers) and in women with PVs in each gene. For these models, breast cancer status was the dependent variable, and independent variables included the risk factor of interest, age at WHI enrollment, and AS.

Reasoning that associations of larger magnitude could have more significance in clinical decision-making, we sought to rule out twofold or greater increases in risk with established risk factors. Accordingly, we calculated one-sided upper 95% CIs to rule out twofold or greater increases in risk for PV carriers due to clinical factors expected to be associated with higher breast cancer risk (BMI, family history of breast cancer, smoking, alcohol consumption, NSES, and MHT), and one-sided lower 95% CIs for clinical factors expected to be associated with lower risk (parity, breastfeeding, oophorectomy, and tubal ligation).

For the secondary analysis, we tested whether established factors modify breast cancer risk differently for PV carriers versus noncarriers by fitting interaction terms in logistic regression models. Models were constructed separately in subcohorts comprised of noncarriers and carriers of PVs in the gene of interest. For each model, breast cancer status was the dependent variable. Independent variables included the risk factor of interest, PV status for the gene of interest, age at WHI enrollment, study, and a term for “risk factor × PV” interaction.

In an exploratory analysis, we reasoned that the effects of risk factors might differ by the estrogen responsiveness of breast cancers to which PV carriers are predisposed. Thus, we repeated the analyses described above by groups of genes according to association with estrogen receptor (ER)-positive breast cancer (ER-positive genes: *ATM* and *CHEK2*, which are not significantly associated with ER-negative disease) and ER-negative breast cancer (ER-negative genes: *BRCA1* and *PALB2*, with ORs for ER-negative disease at least double those for ER-positive disease). These associations were based on results of the large CARRIERS and BRIDGES studies (4, 29), not on the ER status of tumors that developed in participants of AS508 or AS551.

### Data availability

The data generated in this study are available upon request from the corresponding author. Sequencing data used in this study are available upon request to the WHI Coordinating Center, in accordance with the WHI policy for accessing genetic data (available at https://www.whi.org/md/gwas).

## Results

A total of 12,957 WHI participants were included: 4,517 comprising AS508 and 8,440 comprising AS551 (Table 1). The median age at WHI enrollment was 63 years. The racial distribution was 80.6% White in AS508 and 99.0% White in AS551, for an overall distribution of 92.6% White, 4.0% Black, 1.4% Asian, 0.2% American Indian or Alaskan Native, <0.1% Native Hawaiian or Pacific Islander, 1.1% with two or more races, and 0.7% with unknown or not reported race. The ethnic distribution was 5.4% Spanish/Hispanic/Latina in AS508 and 0.5% Spanish/Hispanic/Latina in AS551, for an overall distribution of 2.2% Spanish/Hispanic/Latina, 97.7% not Spanish/Hispanic/Latina, and 0.2% missing data. The BMI was 29.2% obese and 34.6% overweight; 16.6% had a first-degree relative with breast cancer. Approximately half of participants ever smoked, most consumed some alcohol and were parous, approximately half had breastfed, and 17% to 18% had oophorectomy or tubal ligation. The mean NSES was 76.8 (SD 7.65), similar to that in other WHI studies (26). Slightly less than half (43.8%) of participants never used MHT; 32.6% used E + P MHT (685 in the WHI E + P clinical trial and 3,536 by self-report in the observational study), and 23.6% used E-only MHT (297 in the WHI E-only clinical trial and 2,761 by self-report in the observational study; Table 1).

**Table 1:** Participant characteristics of WHI ASs AS508 and AS551

Characteristic	AS508, *N* = 4,517	AS551, *N* = 8,440	Total, *N* = 12,957
Median age at study enrollment (years)	62	63	63
Race			
White	3,641 (80.6%)	8,358 (99.0%)	11,999 (92.6%)
Black or African American	513 (11.4%)	2 (<0.1%)	515 (4.0%)
Asian	179 (4.0%)	1 (<0.1%)	180 (1.4%)
Native Hawaiian or Pacific Islander	5 (0.1%)	0	5 (<0.1%)
American Indian or Alaskan Native	17 (0.4%)	5 (0.1%)	22 (0.2%)
Two or more races	86 (1.9%)	63 (0.7%)	149 (1.1%)
Unknown/not reported	76 (1.7%)	11 (0.1%)	87 (0.7%)
Ethnicity			
Spanish/Hispanic/Latina	245 (5.4%)	39 (0.5%)	284 (2.2%)
Not Spanish/Hispanic/Latina	4,252 (94.1%)	8,401 (99.5%)	12,653 (97.7%)
Missing	20 (0.4%)	0	20 (0.2%)
BMI			
Underweight (<18.5 kg/m^2^)	22 (0.5%)	57 (0.7%)	79 (0.6%)
Normal (18.5–24.9 kg/m^2^)	1,524 (33.7%)	3,088 (36.6%)	4,612 (35.6%)
Overweight (25–29.9 kg/m^2^)	1,537 (34.0%)	2,945 (34.9%)	4,482 (34.6%)
Obese (≥30 kg/m^2^)	1,431 (31.7%)	2,347 (27.8%)	3,778 (29.2%)
Missing	3 (0.1%)	3 (<0.1%)	6 (<0.1%)
Breast cancer diagnosis			
Yes	2,067 (45.8%)	4,453 (52.8%)	6,520 (50.3%)
ER-positive	1,607 (35.6%)	3,601 (42.7%)	5,208 (40.2%)
ER-negative	295 (6.5%)	560 (6.6%)	855 (6.6%)
Borderline	1 (<0.1%)	8 (0.1%)	9 (0.1%)
Unknown^a^	164 (3.6%)	284 (3.4%)	448 (3.5%)
No	2,450 (54.2%)	3,987 (47.2%)	6,437 (49.7%)
Family history of breast cancer in first-degree relatives			
Yes	692 (15.3%)	1,453 (17.2%)	2,145 (16.6%)
No	3,820 (84.6%)	6,966 (82.5%)	10,786 (83.2%)
Cigarette smoking (ever use)			
Yes	2,060 (45.6%)	4,057 (48.1%)	6,117 (47.2%)
No	2,307 (51.1%)	4,096 (48.5%)	6,403 (49.4%)
Missing	150 (3.3%)	287 (3.4%)	437 (3.4%)
Alcohol consumption (ever use)			
Yes	4,024 (89.1%)	7,701 (91.2%)	11,725 (90.5%)
No	468 (10.4%)	688 (8.2%)	1,156 (8.9%)
Missing	25 (0.6%)	51 (0.6%)	76 (0.6%)
Parity status (any)			
Parous	3,939 (87.2%)	7,370 (87.3%)	11,309 (87.3%)
Nulliparous	551 (12.2%)	1,017 (12.0%)	1,568 (12.1%)
Missing	27 (0.6%)	53 (0.6%)	80 (0.6%)
Breastfeeding history			
Ever	2,273 (50.3%)	4,340 (51.4%)	6,613 (51.0%)
Never	2,184 (48.4%)	4,007 (47.5%)	6,191 (47.8%)
Missing	60 (1.3%)	93 (1.1%)	153 (1.2%)
Oophorectomy			
Yes	911 (20.2%)	1,467 (17.4%)	2,378 (18.4%)
No	3,507 (77.6%)	6,837 (81.0%)	10,344 (79.8%)
Missing	99 (2.2%)	136 (1.6%)	235 (1.8%)
Tubal ligation			
Yes	889 (19.7%)	1,384 (16.4%)	2,273 (17.5%)
No	3,609 (79.9%)	7,006 (83.0%)	10,615 (81.9%)
Missing	19 (0.4%)	50 (0.6%)	69 (0.5%)
NSES			
Mean (SD)	75.6 (8.73)	77.5 (6.93)	76.8 (7.65)
MHT			
E + P	1,307 (28.9%)	2,914 (34.5%)	4,221 (32.6%)
Received on the E + P randomized trial treatment arm^b^	225 (5.0%)	460 (5.5%)	685 (5.3%)
Self-report of use (not enrolled in the E + P trial)	1,082 (24.0%)	2,454 (29.1%)	3,536 (27.3%)
E-only	1,119 (24.8%)	1,939 (23.0%)	3,058 (23.6%)
Received on the E-only randomized trial treatment arm^b^	127 (2.8%)	170 (2.0%)	297 (2.3%)
Self-report of use (not enrolled in the E-only trial)	992 (22.0%)	1,769 (21.0%)	2,761 (21.3%)
Never used	2,091 (46.3%)	3,587 (42.5%)	5,678 (43.8%)
Randomized to the placebo arm, E + P trial	161 (3.6%)	310 (3.7%)	471 (3.6%)
Randomized to the placebo arm, E-only trial	119 (2.6%)	160 (1.9%)	279 (2.2%)
Self-report of never use (not enrolled in trial)	1,811 (40.1%)	3,117 (36.9%)	4,928 (38.0%)
Dietary modification clinical trial enrollment			
Yes	1,468 (32.5%)	2,619 (31.0%)	4,087 (31.5%)
No	3,049 (67.5%)	5,821 (69.0%)	8,870 (68.5%)
Calcium and vitamin D clinical trial enrollment			
Yes	1,088 (24.1%)	1,923 (22.8%)	3,011 (23.2%)
No	3,429 (75.9%)	6,517 (77.2%)	9,946 (76.8%)
Observational study enrollment			
Yes	2,550 (56.5%)	5,014 (59.4%)	7,564 (58.4%)
No	1,967 (43.5%)	3,426 (40.6%)	5,393 (41.6%)

a Results unavailable, unknown, or missing.

b If a participant was randomized to the placebo arm of either the E + P MHT clinical trial or the E-only MHT clinical trial, we used that participant’s self-reported data to categorize her MHT use for analyses. Thus, we categorized 69 women assigned to the E-only MHT trial placebo arm into E + P (*n* = 12) or E-only (*n* = 57) use and 61 women assigned to the E + P MHT trial placebo arm into E + P (*n* = 54) or E-only (*n* = 7) use.

PV prevalence was as follows: *BRCA1*, *n* = 34, 0.26%; *BRCA2*, *n* = 62, 0.48%; *ATM*, *n* = 65, 0.50%; *CHEK2*, *n* = 93, 0.72%; and *PALB2*, *n* = 33, 0.25%. Participant characteristics according to PV status are shown in Supplementary Table S1. Penetrance was as follows: *BRCA1*, OR = 4.87 (95% CI, 2.11–13.60); *BRCA2*, OR = 4.71 (95% CI, 2.57–9.41); *ATM*, OR = 2.24 (95% CI, 1.34–3.87); *CHEK2*, OR = 2.05 (95% CI, 1.34–3.21); and *PALB2*, OR = 6.94 (95% CI, 2.83–21.78; Table 2).

**Table 2:** Prevalence and penetrance of *BRCA1*, *BRCA2*, *ATM*, *CHEK2*, and *PALB2* PVs

Affected gene^a^	Prevalence, AS 508	Prevalence, AS 551	Prevalence, total	Penetrance^b^
	*N* = 4,517	*N* = 8,440	*N* = 12,957	*N* = 12,957
	*n* (%)	*n* (%)	*n* (%)	OR (95% CI)
*BRCA1*	8 (0.18)	26 (0.31)	34 (0.26)	4.87 (2.11–13.60)
*BRCA2*	23 (0.51)	39 (0.46)	62 (0.48)	4.71 (2.57–9.41)
*ATM*	27 (0.60)	38 (0.45)	65 (0.50)	2.24 (1.34–3.87)
*CHEK2* ^c^	20 (0.44)	73 (0.86)	93 (0.72)	2.05 (1.34–3.21)
*PALB2*	16 (0.35)	17 (0.20)	33 (0.25)	6.94 (2.83–21.76)

a Three participants had PVs in two genes: one in *BRCA1* and *CHEK2*, one in *BRCA2* and *PALB2*, and one in *CHEK2* and *PALB2*.

b Penetrance is expressed as the OR of developing breast cancer among PV carriers vs. participants testing negative using a multivariable logistic regression model controlling for AS (508 vs. 551) and age at enrollment.

c The difference in the number of *CHEK2* PVs between the ASs was due in part to the *CHEK2* 1100delC variant, a common founder variant in people of European ancestry which was found in 46 participants in AS551 vs. 8 in AS508.

Table 3 shows results from multivariable models of the increase in the odds of developing breast cancer associated with each risk factor among noncarriers and carriers of PVs. We observed modest evidence of reduced breast cancer risk associated with breastfeeding for carriers of PVs in *PALB2* (OR = 0.08; 95% CI, 0.00–0.92; *P* value = 0.042). When examining individual genes, statistical power was limited due to the small number of PV carriers. However, power was sufficient to rule out a twofold or greater increase in the odds of developing breast cancer based on one-sided 95% CIs for the following risk factors: obesity for *ATM* or *BRCA2* carriers; smoking and alcohol for *CHEK2* carriers; not breastfeeding for *ATM* carriers; not having oophorectomy for *BRCA2* or *CHEK2* carriers; not having tubal ligation for *CHEK2* carriers; and NSES for carriers of PVs in any gene.

**Table 3:** Multivariable models of the odds of developing breast cancer in carriers of PVs by risk factor

	*BRCA1*	*BRCA2*	*ATM*	*CHEK2*	*PALB2*	Negative test^a^
Risk factor	OR (95% CI)	OR (95% CI)	OR (95% CI)	OR (95% CI)	OR (95% CI)	OR (95% CI)
BMI (obese vs. normal)	0.68 (0.08–5.67)	0.49 (0.10–2.35)^b^	0.67 (0.18–2.45)^b^	1.71 (0.60–5.02)	1.02 (0.12–12.67)	1.33 (1.22–1.45)***
Family history (FDR vs. none)	0.48 (0.07–2.80)	1.60 (0.39–9.05)	1.48 (0.42–6.01)	1.11 (0.39–3.42)	1.78 (0.23–23.05)	1.42 (1.29–1.57)***
NSES (per SD)	0.95 (0.54–1.45)^b^	1.17 (0.61–2.15)^b^	1.20 (0.66–2.21)^b^	0.84 (0.57–1.21)^b^	0.89 (0.36–1.44)^b^	0.97 (0.93–1.00)
Smoking (ever vs. never)	2.59 (0.40–29.14)	1.51 (0.39–6.83)	0.89 (0.29–2.73)	0.47 (0.18–1.18)^b^	1.97 (0.25–22.17)	1.11 (1.04–1.20)**
Alcohol (ever vs. never)	2.58 (0.16–39.90)	1.22 (0.11–7.64)	1.70 (0.24–11.23)	0.61 (0.14–2.19)^b^	0.72 (0.01–9.28)	1.01 (0.90–1.15)
Parity (parous vs. nulliparous)	4.66 (0.30–72.55)	0.27 (0.00–2.81)	0.42 (0.04–2.84)	0.44 (0.10–1.56)	0.32 (0.00–4.71)	0.81 (0.72–0.90)***
Breastfeeding (ever vs. never)	1.24 (0.11–8.53)	1.13 (0.30–4.22)	2.01 (0.63–6.86)^c^	0.53 (0.21–1.28)	0.08 (0.00–0.92)*	1.02 (0.95–1.09)
Oophorectomy (yes vs. no)	0.69 (0.09–5.24)	3.07 (0.56–32.56)^c^	0.97 (0.22–4.92)	1.68 (0.57–5.68)^c^	1.02 (0.05–156.78)	0.83 (0.76–0.91)***
Tubal ligation (yes vs. no)	0.73 (0.07–9.86)	0.86 (0.17–5.77)	0.61 (0.13–3.03)	2.40 (0.61–13.62)^c^	0.80 (0.11–8.98)	0.96 (0.87–1.06)
MHT (E + P)	N/A	N/A	6.18 (0.23–5.7 × 10^4^)	6.96 (0.74–172.88)	N/A	1.35 (1.06–1.74)*
MHT (E-only)	N/A	N/A	3.4 (0.00–145.68)	2.74 (0.29–45.47)	N/A	0.93 (0.69–1.24)

*, *P* < 0.05; **, *P* < 0.01; and ***, *P* < 0.001.

Abbreviations: FDR, first-degree relative; N/A, not applicable (too few participants to analyze).

a A greater than twofold change in the odds of developing breast cancer is ruled out for all risk factors among women with negative tests.

b A greater than twofold increase in the odds of developing breast cancer associated with this risk factor is ruled out for this gene, based on one-sided 95% upper CI limits.

c A greater than twofold decrease in the odds of developing breast cancer associated with this risk factor is ruled out for this gene, based on one-sided 95% lower CI limits.

Table 4 shows results from multivariable models of the exploratory analysis of the increase in the odds of developing breast cancer associated with each risk factor among women who carry PVs in ER-positive breast cancer risk genes (*ATM* and *CHEK2*) and ER-negative breast cancer risk genes (*BRCA1* and *PALB2*). The results suggest that E + P MHT use may be associated with high breast cancer risk in women who carry PVs in ER-positive genes (OR = 7.31; 95% CI, 1.14–64.20; *P* value = 0.036). Power was sufficient to exclude a twofold or greater increase in the odds of developing breast cancer due to smoking, alcohol consumption, and not having an oophorectomy or tubal ligation for ER-positive PV carriers and due to NSES for both ER-negative and ER-positive gene PV carriers.

**Table 4:** Exploratory analysis using multivariable models of the increase in the odds of developing breast cancer in carriers of PVs in genes grouped by ER status of associated cancers by risk factor

	ER-positive genes (*ATM* and *CHEK2*)	ER-negative genes (*BRCA1* and *PALB2*)	Negative test^a^
Risk factor	OR (95% CI)	OR (95% CI)	OR (95% CI)
BMI (obese vs. normal)	1.16 (0.51–2.65)	0.81 (0.17–4.20)	1.33 (1.22–1.45)***
Family history (FDR vs. none)	1.26 (0.56–2.99)	0.84 (0.22–3.49)	1.42 (1.29–1.57)***
NSES (per SD)	0.96 (0.67–1.34)^b^	0.91 (0.58–1.28)^b^	0.97 (0.97–1.00)
Smoking (ever vs. never)	0.60 (0.29–1.22)^b^	2.50 (0.59–14.30)	1.11 (1.04–1.20)**
Alcohol (ever vs. never)	0.79 (0.24–2.32)^b^	1.38 (0.13–8.38)	1.01 (0.90–1.15)
Parity (ever vs. never)	0.40 (0.11–1.21)	1.05 (0.09–6.63)	0.81 (0.72–0.90)***
Breastfeeding (ever vs. never)	0.85 (0.41–1.71)	0.26 (0.03–1.31)	1.02 (0.95–1.09)
Oophorectomy	1.38 (0.58–3.56)^c^	0.69 (0.17–3.25)	0.83 (0.76–0.91)***
Tubal ligation	1.39 (0.51–4.35)^c^	0.84 (0.18–5.12)	0.96 (0.87–1.06)
MHT (E + P)	7.31 (1.14–64.20)*	0.68 (0.07–9.07)	1.35 (1.06–1.74)*
MHT (E-only)	2.07 (0.28–15.26)	1.33 (0.13–18.74)	0.93 (0.69–1.24)

*, *P* < 0.05; **, *P* < 0.01; and ***, *P* < 0.001.

Abbreviation: FDR, first-degree relative.

a A greater than twofold change in the odds of developing breast cancer is ruled out for all risk factors among women with negative tests.

b A greater than twofold increase in the odds of developing breast cancer associated with this risk factor is ruled out for this gene, based on one-sided 95% upper CI limits.

c A greater than twofold decrease in the odds of developing breast cancer associated with this risk factor is ruled out for this gene, based on one-sided 95% lower CI limits.

At the 5% significance level, we found no evidence supporting an interaction of clinical risk factors with PVs in any gene or group of genes (all *P* values for interaction ≥0.05). The strongest results from interaction testing suggested a possibly greater risk reduction due to breastfeeding for *PALB2* carriers versus noncarriers (*P* value for interaction = 0.053) and a potentially greater increase in risk due to E + P MHT for ER-positive gene carriers versus noncarriers (*P* value for interaction = 0.094).

## Discussion

We characterized breast cancer risk associated with established risk factors among WHI participants who carry PVs in *BRCA1/2*, *ATM*, *CHEK2*, and *PALB2*. There was evidence of reduced breast cancer risk with breastfeeding in *PALB2* PV carriers, which may offer a practical option for risk modification. Whereas statistical power was limited, it was sufficient to rule out a twofold or greater increase in breast cancer risk for PV carriers due to obesity, alcohol consumption, tobacco use, and NSES. These results should in no way discourage public health messages to modify these risk factors but may offer reassurance that exposure does not substantially increase breast cancer risk (by twofold or higher) among PV carriers; however, more modest increases could not be ruled out. Moreover, in an exploratory analysis of grouping genes that were previously reported to predispose toward ER-positive disease, use of E + P MHT was modestly associated with high breast cancer risk in women with *ATM* or *CHEK2* PVs.

Prior studies have focused on *BRCA1/2* PV carriers and generally shown little or no increase in breast cancer risk associated with alcohol consumption, tobacco use, or obesity (7). Such prior findings contrast with those for the general population, in which alcohol consumption has been associated with relative risks in range of 1.1 to 1.2 (for one drink per day), tobacco use in range of 1.2 to 1.5, and postmenopausal obesity in range of 1.2 (30, 31). High parity has been associated with a lower risk of breast cancer (in range of 0.7) among *BRCA1/2* PV carriers, and breastfeeding with a lower risk (in range of 0.6) among *BRCA1* PVs carriers (32, 33), similar to the lower risks associated with high parity (in range of 0.7) and breastfeeding (in range of 0.8) in the general population (30, 31). Premenopausal salpingo-oophorectomy, recommended for *BRCA1/2* PV carriers given their high ovarian cancer risk, has been variably associated with lower breast cancer risk (in range of 0.2–0.9) depending on the affected gene and age at surgery (34) and aligns with general population findings. MHT after premenopausal salpingo-oophorectomy in *BRCA1/2* PV carriers has generally not been associated with higher breast cancer risk (35). Although this study lacked power to detect risk increases larger than twofold, its findings are largely consistent with prior reports, suggesting that most established breast cancer risk factors do relatively little to modify breast cancer risk in *BRCA1/2* PV carriers.

Whereas studies have found little modification of *BRCA1/2*-associated risks, there has been evidence of greater risk modification with moderate penetrance genes, notably *CHEK2* (36). Given the small number of PVs, we jointly analyzed two moderate penetrance genes, *ATM* and *CHEK2*, due to their shared association with ER-positive breast cancer in prior studies (4, 29); the result of this exploratory analysis was an increase in breast cancer risk (OR = 7.3) associated with E + P MHT. These findings should be considered hypothesis-generating, given their wide CI. However, they concur with the WHI randomized clinical trial results that showed higher breast cancer risk with E + P but not E-only MHT (37). Unlike *BRCA1/2*, PVs in *ATM* and *CHEK2* do not confer sufficient ovarian cancer risk to warrant recommendation of risk-reducing salpingo-oophorectomy. Thus, MHT after natural menopause (as was studied in WHI) may be a clinically relevant option for *ATM* or *CHEK2* PV carriers, and its potential impact on breast cancer risk should be further studied. Notably, however, WHI used a single dose of combined E + P for all clinical trial participants, which may not reflect the needs of women in different stages of menopause and differs from regimens typically used nowadays (38). Research on the potential interaction of modern MHT regimens with breast cancer risk due to PVs in *ATM*, *CHEK2*, and other genes should be prioritized.

This study has some limitations. As noted previously, the number of PV carriers was small, limiting statistical power. The WHI enrolled only women who had not developed breast cancer before menopause: thus, the findings may not apply to younger PV carriers. The sample had limited racial and ethnic diversity and, given its enrollment from 1993 to 1998, may not reflect the exposures, reproductive experiences, or, as noted above, MHT regimens of more recent birth cohorts. Study strengths include a large, well-characterized sample within the prospective WHI; more than 20 years of follow-up time; and confirmation of all cancer diagnoses, with careful ascertainment of risk factors, including type of MHT.

In summary, postmenopausal carriers of PVs in *BRCA1/2*, *ATM*, *CHEK2*, and *PALB2* are unlikely to have substantial (twofold or greater) elevations in the risk of developing breast cancer due to reproductive and lifestyle exposures. However, additional research is warranted to determine whether MHT modifies the risk conferred by PVs in breast cancer susceptibility genes.

## Supplementary Material

Supplementary Methods Supplementary methods

Supplementary Table 1 Supplementary Table 1
