# Supplementary material for: Breast Cancer Risk Modification in Women with Pathogenic Variants in BRCA1, BRCA2, ATM, CHEK2, and PALB2
Source: Cancer Res Commun. 2025 May 12;5(5):783–91. doi: 10.1158/2767-9764.CRC-24-0592 (PMC12067184; doi:10.1158/2767-9764.CRC-24-0592)
Supplement: Supplementary Methods [file crc-24-0592_supplementary_methods_suppsm.pdf]

Supplementary Methods

Coding of Variables in Logistic Regression Analysis

This study employed logistic regression models with Firth's penalized likelihood[1]. Breast cancer status was coded as binary (affected versus unaffected) and used as the dependent variable in every model. All models included independent variables for age at WHI enrollment (in years) as a quantitative variable and ancillary study (AS508 versus AS551) as a binary variable.

Pathogenic variant (PV) status was coded for each gene as a binary variable (PV-positive versus PV-negative). Patients with a genetic variant classified as deleterious or suspected deleterious were coded as PV-positive. Patients were coded as PV-negative if only benign polymorphisms or no variants were detected. Patients with variants of unknown significance (VUS) were excluded from the analysis of the gene in question unless they also had a PV in that gene.

Risk factors at WHI enrollment included body mass index (BMI), family history of breast cancer, smoking, alcohol consumption, parity, breastfeeding, oophorectomy and tubal ligation status, neighborhood socioeconomic status (NSES), and menopausal hormone therapy (MHT) use of estrogen and progestin (E+P) or estrogen only (E-only). BMI was coded as a 4-level categorical variable (underweight, <18.5 kg/m^2; normal, 18.5-24.9 kg/m^2; overweight, 25-29.9 kg/m^2; obese, ≥30 kg/m^2). Family history of breast cancer in one or more first-degree relatives was coded as binary (yes/no). Smoking and alcohol consumption were each coded as binary (ever/never). Parity was coded as binary

(parous/nulliparous). Breastfeeding was coded as binary (ever/never). Oophorectomy was coded as binary (yes/no). Tubal ligation was coded as binary (yes/no). NSES was coded as a quantitative variable as previously described[2]. MHT was coded as a 3-level categorical variable (never used; E+P; E-only). If a participant was randomized to the placebo arm of either the E+P MHT clinical trial or the E-only MHT clinical trial, we used that participant's self-reported data to categorize her MHT use for analyses. Risk due to HRT is expected to ramp down for two years following cessation, with no increased risk thereafter[3-5]. We, therefore, coded participants who reported MHT cessation two or more years before enrollment as never users, and models of breast cancer risk associated with MHT only considered breast cancers that were diagnosed within two years of reported MHT use.

REFERENCES

1. Firth, David. "Bias reduction of maximum likelihood estimates." *Biometrika* 80.1 (1993): 27-38.

2. Griffin, Beth Ann, et al. "The relationship between urban sprawl and coronary heart disease in women." *Health & Place* 20 (2013): 51-61.

3. Brentnall, Adam R., and Jack Cuzick. "Risk models for breast cancer and their validation." *Statistical Science: A Review Journal of the Institute of Mathematical Statistics* 35.1 (2020): 14.

4. Chlebowski, Rowan T., and Garnet L. Anderson. "The Influence of Time From Menopause and Mammography on Hormone Therapy–Related Breast Cancer Risk Assessment." *Journal of the National Cancer Institute* 103.4 (2011): 284-285.

5. Reeves, Gillian K., et al. "Hormonal therapy for menopause and breast-cancer risk by histological type: a cohort study and meta-analysis." *The Lancet Oncology* 7.11 (2006): 910-918.
