# Supplementary material for: Breast Cancer Risk Modification in Women with Pathogenic Variants in BRCA1, BRCA2, ATM, CHEK2, and PALB2
Source: Cancer Res Commun. 2025 May 12;5(5):783–91. doi: 10.1158/2767-9764.CRC-24-0592 (PMC12067184; doi:10.1158/2767-9764.CRC-24-0592)
Supplement: Supplementary Table 1 [file crc-24-0592_supplementary_table_1_suppst1.pdf]

**Supplementary Table 1.** Characteristics of participants with pathogenic variants in *BRCA1, BRCA2, ATM, CHEK2* or *PALB2* in WHI AS508 and AS551					
	BRCA1, N=34	*BRCA2*, N=62	*ATM*, N=65	*CHEK2*, N=93	*PALB2*, N=33
**Median Age at Study Enrollment** (years)	58.5	63	61	63	63
**Race**					
White	28 (82.4%)	55 (88.7%)	57 (87.7%)	91 (97.8%)	27 (81.8%)
Black or African American	4 (11.8%)	5 (8.1%)	6 (9.2%)	1 (1.1%)	5 (15.2%)
Asian	2 (5.9%)	1 (1.6%)	1 (1.5%)	0	0
Native Hawaiian or Pacific Islander	0	0	0	0	0
American Indian or Alaskan Native	0	0	0	0	0
Two or more races	0	0	1 (1.5%)	1 (1.1%)	0
Unknown/Not reported	0	1 (1.6%)	0	0	1 (3.0%)
**Ethnicity**					
Spanish/Hispanic/Latina	0	2 (3.2%)	0	0	3 (9.1%)
Not Spanish/Hispanic/Latina	34 (100%)	60 (96.8%)	65 (100%)	93 (100%)	30 (90.9%)
Missing	0	0	0	0	0
**Body Mass Index**					
Underweight (<18.5 kg/m^2)	1 (2.9%)	0	0	0	0
Normal (18.5-24.9 kg/m^2)	12 (35.3%)	24 (38.7%)	25 (38.5%)	32 (34.4%)	14 (42.4%)
Overweight (25-29.9 kg/m^2)	13 (38.2%)	25 (40.3%)	18 (27.7%)	30 (32.3%)	11 (33.3%)
Obese (≥30 kg/m^2)	8 (23.5%)	13 (21%)	22 (33.8%)	31 (33.3%)	8 (24.2%)
Missing	0	0	0	0	0
**Breast Cancer Diagnosis**					
Yes	29 (85.3%)	51 (82.3%)	45 (69.2%)	63 (67.7%)	29 (87.9%)
ER-Positive	7 (20.6%)	38 (61.3%)	38 (58.5%)	50 (53.8%)	24 (72.7%)
ER-Negative	18 (52.9%)	12 (19.4%)	2 (3.1%)	5 (5.4%)	5 (15.2%)
Borderline	0	0	0	0	0
Unknown*	4 (11.8%)	1 (1.6%)	5 (7.7%)	8 (8.6%)	0
No	5 (14.7%)	11 (17.7%)	20 (30.8%)	30 (32.3%)	4 (12.1%)
**Family History of breast cancer in first-degree relatives**					
Yes	15 (44.1%)	18 (29.0%)	17 (26.2%)	19 (20.4%)	12 (36.4%)
No	19 (55.9%)	44 (71.0%)	48 (73.8%)	74 (79.6%)	21 (63.6%)
**Cigarette Smoking** (ever use)					
Yes	13 (38.2%)	26 (41.9%)	28 (43.1%)	46 (49.5%)	17 (51.5%)
No	19 (55.9%)	34 (54.8%)	35 (53.8%)	43 (46.2%)	15 (45.5%)
Missing	2 (5.9%)	2 (3.2%)	2 (3.1%)	4 (4.3%)	1 (3.0%)
**Alcohol consumption** (ever use)					
Yes	31 (91.2%)	55 (88.7%)	59 (90.8%)	80 (86.0%)	30 (90.9%)
No	3 (8.8%)	6 (9.7%)	5 (7.7%)	12 (12.9%)	3 (9.1%)
Missing	0	1 (1.6%)	1 (1.5%)	1 (1.1%)	0
**Parity status** (any)					
Parous	32 (94.1%)	56 (90.3%)	59 (90.8%)	77 (82.8%)	28 (84.8%)
Nulliparous	2 (5.9%)	5 (8.1%)	6 (9.2%)	15 (16.1%)	5 (15.2%)

Missing	0	1 (1.6%)	0	1 (1.1%)	0
**Breastfeeding history**					
Ever	27 (79.4%)	30 (48.4%)	32 (49.2%)	47 (50.5%)	18 (54.5%)
Never	7 (20.6%)	28 (45.2%)	32 (49.2%)	45 (48.4%)	15 (45.5%)
Missing	0	4 (6.5%)	1 (1.5%)	1 (1.1%)	0
**Oophorectomy**					
Yes	15 (44.1%)	12 (19.4%)	10 (15.4%)	21 (22.6%)	2 (6.1%)
No	19 (55.9%)	49 (79%)	55 (84.6%)	69 (74.2%)	31 (93.9%)
Missing	0	1 (1.6%)	0	3 (3.2%)	0
**Tubal Ligation**					
Yes	9 (26.5%)	9 (14.5%)	10 (15.4%)	13 (14%)	8 (24.2%)
No	25 (73.5%)	53 (85.5%)	55 (84.6%)	80 (86%)	25 (75.8%)
Missing	0	0	0	0	0
**Neighborhood Socioeconomic Status (NSES)**					
Mean (Standard Deviation)	75.1 (9.05)	77.7 (6.68)	76.5 (7.27)	78.3 (6.43)	76.8 (9.16)
**Menopausal Hormone Therapy (MHT)**					
Estrogen and progestin (E+P)	6 (17.6%)	14 (22.6%)	18 (27.7%)	28 (30.1%)	13 (39.4%)
Received on E+P randomized trial treatment arm**	1 (2.9%)	3 (4.8%)	3 (4.6%)	0	4 (12.1%)
Self-report of use (not enrolled in E+P trial)	5 (14.7%)	11 (17.7%)	15 (23.1%)	28 (30.1%)	9 (27.3%)
Estrogen (E) only	13 (38.2%)	17 (27.4%)	17 (26.2%)	26 (28%)	4 (12.1%)
Received on E-only randomized trial treatment arm**	1 (2.9%)	2 (3.2%)	1 (1.5%)	3 (3.2%)	1 (3.0%)
Self-report of use (not enrolled in E-only trial)	12 (35.3%)	15 (24.2%)	16 (24.6%)	23 (24.7%)	3 (9.1%)
Never used	15 (44.1%)	31 (50%)	30 (46.2%)	39 (41.9%)	16 (48.5%)
Randomized to placebo arm, E+P trial	1 (2.9%)	3 (4.8%)	2 (3.1%)	3 (3.2%)	1 (3.0%)
Randomized to placebo arm, E-only trial	2 (5.9%)	0	3 (4.6%)	1 (1.1%)	0
Self-report of never use (not enrolled in trial)	12 (35.3%)	28 (45.2%)	25 (38.5%)	35 (37.6%)	15 (45.5%)
**Dietary Modification Clinical Trial Enrollment**					
Yes	10 (29.4%)	19 (30.6%)	19 (29.2%)	33 (35.5%)	8 (24.2%)
No	24 (70.6%)	43 (69.4%)	46 (70.8%)	60 (64.5%)	25 (75.8%)
**Calcium and Vitamin D Clinical Trial Enrollment**					
Yes	6 (17.6%)	15 (24.2%)	14 (21.5%)	24 (25.8%)	10 (30.3%)
No	28 (82.4%)	47 (75.8%)	51 (78.5%)	69 (74.2%)	23 (69.7%)
**Observational Study Enrollment**					
Yes	20 (58.8%)	38 (61.3%)	39 (60%)	56 (60.2%)	19 (57.6%)
No	14 (41.2%)	24 (38.7%)	26 (40%)	37 (39.8%)	14 (42.4%)

WHI: Women's Health Initiative; AS, Ancillary Study
*Results Unavailable, Unknown or Missing
**If a participant was randomized to the placebo arm of either the E+P MHT clinical trial or the E-only MHT clinical trial, we used that participant's self-reported data to categorize her MHT use for analyses.
